# Sarcopenia in people living with HIV in Hong Kong: which definition correlates with health outcomes?

**DOI:** 10.1002/jia2.25988

**Published:** 2022-09-29

**Authors:** Fion Wing Lam Luk, Timothy Li, Hang Yee Ho, Yin Yan Chan, Siu King Cheung, Vickie Wong, Timothy Chi Yui Kwok, Grace Lui

**Affiliations:** ^1^ Department of Medicine and Therapeutics Prince of Wales Hospital Sha Tin Hong Kong; ^2^ Department of Medicine and Therapeutics The Chinese University of Hong Kong Ma Liu Shui Hong Kong

**Keywords:** HIV, sarcopenia, quality of life, disability, polypharmacy, ageing

## Abstract

**Introduction:**

Sarcopenia is an important clinical syndrome in older people living with HIV (PLWH). With a change to the Asia sarcopenia definition in 2019, we aimed to determine whether health outcomes were associated with different definitions of sarcopenia among Asian PLWH.

**Methods:**

We performed a prospective cross‐sectional study enrolling PLWH aged ≥35 years from January 2018 to November 2021. We defined sarcopenia by the Asia Working Group of Sarcopenia (AWGS) criteria in 2014 and 2019. AWGS‐2014 included low muscle mass plus weak handgrip strength and/or slow gait speed. AWGS‐2019 included low muscle mass plus low muscle strength or physical performance, while the presence of all defines severe sarcopenia. We measured appendicular skeletal muscle mass using dual‐energy X‐ray absorptiometry, handgrip strength, usual gait speed, five‐time chair stand test and Short Physical Performance Battery. Correlations between each sarcopenia definition and health‐related quality of life (using EQ‐5D‐5L and SF‐36) and functional disability were determined.

**Results:**

One hundred and fifty Asian PLWH were enrolled, 132 (88%) were male, mean age was 60±10 years, duration of HIV diagnosis was 13 (IQR 8–18) years and current CD4 count was 574 (IQR 362–762) cells/mm^3^, 67 (45%) had multimorbidity, 64 (43%) had polypharmacy. Prevalence of sarcopenia by AWGS‐2014, AWGS‐2019 and severe sarcopenia was 17.3%, 27.3% and 18.0%, respectively. Age, education and polypharmacy were associated with sarcopenia. Sarcopenia (AWGS‐2014) and severe sarcopenia were associated with mobility, physical functioning and physical component score (SF‐36). All three criteria were associated with impaired instrumental activities of daily living (IADL). After age and sex adjustment, sarcopenia (AWGS‐2014) (adjusted odds ratio/aOR 5.4, 95% confidence interval/CI 2.0–15.1) and severe sarcopenia (aOR 5.1, 95% CI 1.9–14.0) were associated with mobility and physical component score (SF‐36) (β coefficients –5.3342, *p* = 0.022 and –5.412, *p* = 0.019). Sarcopenia (AWGS 2014) (aOR 5.2, 95% CI 1.7–16.2), sarcopenia (AWGS‐2019) (aOR 4.5, 95% CI 1.5–13.1) and severe sarcopenia (aOR 3.5, 95% CI 1.1–10.9) were associated with impaired IADL in fully adjusted models.

**Conclusions:**

In a sample of Asian PLWH, 17.3%, 27.3% and 18.0% had sarcopenia as defined by AWGS‐2014, AWGS‐2019 and severe sarcopenia, respectively. Sarcopenia by AWGS‐2014 and severe sarcopenia correlated with parameters of poor health outcomes, while sarcopenia by AWGS‐2019 correlated with functional disability.

## 1 INTRODUCTION

Sarcopenia, deriving from the Greek words *sarx* (meaning “flesh”) and *penia* (meaning “loss”), was proposed by Irwin Rosenberg in 1988 to describe an age‐related loss of muscle mass and function [1]. Sarcopenia predicted functional decline, disability, hospitalization, mortality and increased health costs in older adults in the general population [2, 3, 4]. The prevalence of sarcopenia in the general older adult population ranged from 10% to 30% [2].

Sarcopenia is appreciated to be an important phenomenon among older people living with HIV (PLWH) [5]. The pooled prevalence of sarcopenia among PLWH was 24% in a recent meta‐analysis, and PLWH had a six‐fold increased risk of sarcopenia compared with matched HIV‐uninfected controls [6]. Among PLWH, sarcopenia was associated with demographic and HIV‐related factors, including age, sex, education, employment status, smoking, alcohol use, longer duration of HIV infection, CD4 count and longer exposure to anti‐retroviral therapy [6, 7, 8]. Among PLWH, sarcopenia was associated with functional impairment, disability and mortality risk [8, 9]. At present, there is a paucity of data evaluating the prevalence and risk factors of sarcopenia among Asian PLWH [6, 8].

Since the development of the first consensus definition of sarcopenia in 2009 [2], definitions of sarcopenia have evolved as new evidence emerged. In 2010, the European Working Group on Sarcopenia in Older People (EWGSOP1) defined sarcopenia as low muscle mass together with either low muscle strength or low physical performance [10]. A revised version in 2018 (EWGSOP2) focused on low muscle strength, while confirming the diagnosis by low muscle quantity or quality, and defining severe sarcopenia with the additional presence of poor physical performance [11]. In 2014, the Asia Working Group for Sarcopenia (AWGS‐2014) defined sarcopenia as low muscle mass plus either weak handgrip strength or slow gait speed [12]. An updated version in 2019 (AWGS‐2019) retained the original definition of “loss of skeletal muscle mass plus loss of muscle strength and/or reduced physical performance” and added the category of “severe sarcopenia,” which included all three components [13]. In AWGS‐2019, the thresholds for handgrip strength and gait speed were lowered, measures of physical performance were expanded to include a Short Physical Performance Battery (SPPB) and a five‐time chair stand test [12, 13].

Currently, there are no data comparing the AWGS‐2014 and AWGS‐2019 definitions of sarcopenia among PLWH in Asia. It is uncertain which definition of sarcopenia would be more applicable in PLWH. Therefore, we performed this study to determine the prevalence and risk factors of sarcopenia among PLWH living in Hong Kong SAR, China, and the correlation between different definitions of sarcopenia and health outcomes.

## 2 METHODS

### 2.1 Study design and setting

We performed an observational cross‐sectional study involving PLWH recruited from January 2018 to November 2021 at the Prince of Wales Hospital Infectious Diseases clinic in Hong Kong. This clinic received referrals from other HIV clinics for screening or management of chronic comorbidities in PLWH. Inclusion criteria were positive HIV antibody and age of 35 years old or above. The only exclusion criterion was the refusal to consent. The study protocol was approved by the Joint Chinese University of Hong Kong—New Territories East Cluster Clinical Research Ethics Committee.

### 2.2 Data collection

We recorded demographic and clinical information for all participants at each study visit. This included date of birth, sex, smoking, alcohol intake, comorbidities included in Charlson's Comorbidity Index, hepatitis B and C co‐infection, year of diagnosis of HIV, history of AIDS‐defining illness, current and previous anti‐retroviral drug regimens, medications other than anti‐retroviral drugs, current CD4 count, CD4:CD8 ratio and HIV RNA level. We defined multimorbidity as the presence of two or more chronic comorbidities, and polypharmacy as taking five or more non‐anti‐retroviral drugs.

During the study visit, we measured handgrip strength, gait speed, chair stand test and SPPB. We measured handgrip strength three times for both hands, using Jamar Hydraulic Hand dynamometer (5030J1, Sammons Preston, Bolingbrook, IL), with the participant sitting with 90° elbow flexion. The best performance of all trials was used for analyses. We calculated gait speed by measuring the average time of two trials taken to walk 6 metres at a normal pace, using a stop‐watch. We performed the chair stand test by measuring the time taken to perform five chair stands. We performed SPPB, which included the ability to stand for up to 10 seconds with feet positioned in side‐by‐side, semi‐tandem and tandem positions, usual gait speed and five‐time chair stand test [14]. All participants underwent dual‐energy X‐ray absorptiometry using the Hologic QDR 4500A fan‐beam densitometer (Hologic, Inc., Bedford, MA) to measure appendicular skeletal muscle mass, which was adjusted by body height.

### 2.3 Definition of sarcopenia

We defined sarcopenia using AWGS‐2014 and AWGS‐2019 criteria. AWGS‐2014 defines sarcopenia as the presence of low muscle mass (height‐adjusted muscle mass <7 kg/m^2^ in men and <5.4 kg/m^2^ in women) plus weak handgrip strength (<25 kg in men and <18 kg in women) and/or slow gait speed (≤0.8 m/second) [12]. AWGS‐2019 defines sarcopenia as low muscle mass (cut‐offs as above) plus low muscle strength or physical performance, while the presence of all three components defines severe sarcopenia. Low muscle strength is defined as handgrip strength <28 kg for men and <18 kg for women, and low physical performance as gait speed <1 m/second, SPPB score ≤9 or five‐time chair stand test ≥12 seconds [13].

### 2.4 Outcome measures

We measured the following health outcomes: health‐related quality of life (QOL) and functional disability. We measured health‐related QOL by the EuroQOL 5‐dimensional 5‐Level questionnaire (EQ‐5D‐5L) and 36‐item Short Form Health Survey (SF‐36). The EQ‐5D‐5L questionnaire contains five dimensions of QOL: mobility, self‐care, usual activities, pain/discomfort and anxiety/depression. Each of these dimensions is graded as “no problem,” “slight problem,” “moderate problem,” “severe problem” and “extreme problem.” Participants with any severity of the problem in a particular dimension were considered as having a problem with that dimension. The EQ‐5D‐5L index was converted from the EQ‐5D‐5L health states using an established Hong Kong value set by weighting each participant's self‐report health status to a single preference‐based health index [15]. The EuroQOL Visual Analogue Scale (EQ‐VAS) is the self‐reported overall health perception of the participants, recording their self‐rated health on a vertical scale from 0 (the worst health) to 100 (the best health) scale.

Participants also completed the Chinese (Hong Kong) version of SF‐36 [16], which covers eight health domains, including physical function, role limitations related to physical health, bodily pain, mental health, and role limitations related to emotional health, social functioning, vitality and general health. The eight domain scores were summarized into two summary scores: the physical component summary (PCS) and the mental component summary scores. Higher scores indicate better QOL.

Functional disability was defined as an impairment in any domains of activities of daily living (ADL) or instrumental ADL (IADL) [17]. We measured ADL by means of the Katz Index [18] and IADL by Lawton Instrumental Activities of Daily Living (IADL) [19]. The Katz Index assesses dependence or independence concerning six essential functions of ADL (feeding, continence, bathing, transferring, toileting and dressing). Each domain is scored from 0 (independence) to 6 (total dependence). The Lawton IADL scale measures eight domains of function (ability to use telephone, shopping, food preparation, housekeeping, laundry, mode of transportation, responsibility for own medications and the ability to handle finances). Requirement of any assistance in any domain was considered as having impairment in ADL or IADL.

### 2.5 Statistical analysis

We presented descriptive statistics as number (percentage), mean ± standard deviation or median (interquartile range/IQR), as appropriate. We determined agreement between the different definitions of sarcopenia and severe sarcopenia using Cohen's kappa test. Kappa was interpreted as poor if value was < 0.20, fair if 0.21–0.4, moderate if 0.41–0.60, good if 0.61–0.80 and very good if 0.81–1.00 [20]. We compared baseline demographic and clinical variables between those with and without sarcopenia or severe sarcopenia according to different definitions using chi‐square, Student's *t* and Mann–Whitney U test, as appropriate. Age, sex and variables identified to be associated with each definition of sarcopenia in the above univariate analyses were included in multivariable binary logistic stepwise regression models, to determine the variables that were independently associated with each definition of sarcopenia and severe sarcopenia. For each regression analysis, collinearity was assessed using the variance inflation factor.

Associations between different definitions of sarcopenia or severe sarcopenia and disability and health‐related QOL measures were determined using the chi‐square test and Student's *t*‐test, as appropriate. The logistic regression model for binary outcome measure and linear regression model for continuous outcome measure were used to determine the association between sarcopenia and these outcomes in multivariable models. The associations between each definition of sarcopenia and the outcome measures were determined in two multivariable models: model 1 adjusted for age and sex, and model 2 adjusted for age, sex and variables with significant independent association with each definition of sarcopenia. Correlation between different components included in the definitions of sarcopenia and disability and health‐related QOL measures were determined using logistic regression for binary outcome measures and linear regression analyses for continuous outcome measures. Statistical significance was defined as a *p*‐value less than 0.05. Statistical analyses were conducted using SPSS (IBM version 26).

## 3 RESULTS

One hundred and fifty‐six PLWH who were followed up in the clinic were invited to participate in this study, six refused to consent, and 150 participants were recruited. The mean age was 60±10 years, 88.0% were male, 98.7% were Chinese and the median (IQR) duration of HIV diagnosis was 13 [8, 9, 10, 11, 12, 13, 14, 15, 16, 17, 18] years. All participants were receiving antiretroviral therapy, with a current CD4 count of 574 (362–762) cells/mm^3^, and 94% having undetectable HIV RNA (<20 copies/ml). Multimorbidity and polypharmacy were present in 45% and 43% of participants, respectively. The baseline demographic and clinical characteristics are shown in Table 1.

**Table 1:** Baseline characteristics of all participants and participants with sarcopenia

		AWGS‐2014			AWGS‐2019					
Variables	All participants *N* = 150	Sarcopenia *n* = 26	No sarcopenia *n* = 124	*p* ^a^	Sarcopenia *n* = 41	No sarcopenia *n* = 109	*p* ^b^	Severe sarcopenia *n* = 27	No severe sarcopenia *n* = 123	*p* ^c^
Age (years)^d^	60.3 ± 9.6	69.4 ± 7.6	58.4 ± 8.8	<0.001*	66.6 ± 8.9	57.9 ± 8.7	<0.001*	69.6 ± 7.8	58.3 ± 8.7	<0.001*
Male^e^	132 (88.0%)	22 (84.6%)	110 (88.7%)	0.519	35 (85.4%)	97 (89.0%)	0.577	23 (85.2%)	109 (88.6%)	0.743
Smoker^e^	22 (14.7%)	5 (19.2%)	17 (13.7%)	0.541	9 (22.0%)	13 (11.9%)	0.122	7 (25.9%)	15 (12.2%)	0.078
Alcohol intake ≥twice per month^e^	19 (12.7%)	3 (11.5%)	16 (12.9%)	1.000	4 (9.8%)	15 (13.8%)	0.511	3 (11.1%)	16 (13.0%)	1.000
Secondary education or above^e^	120 (80.0%)	12 (46.2%)	108 (87.1%)	<0.001*	26 (63.4%)	94 (86.2%)	0.002	14 (51.9%)	106 (86.2%)	<0.001
No employment^e^	71 (47.3%)	19 (73.1%)	52 (41.9%)	0.004	26 (63.4%)	45 (41.3%)	0.016	20 (74.1%)	51 (41.5%)	0.002
Duration of HIV diagnosis (years)^f^	12.7 (7.6–17.9)	13.4 (7.6–19.5)	12.7 (7.6–17.8)	0.827	15.8 (9.7–19.7)	12.3 (7.3–16.7)	0.044	14.4 (8.9–19.1)	12.7 (7.6–17.7)	0.430
History of AIDS‐defining illness^e^	62 (41.3%)	13 (50.0%)	49 (39.5%)	0.324	18 (43.9%)	44 (40.4%)	0.695	14 (51.9%)	48 (39.0%)	0.220
Current CD4 count (cells/mm^3^)^f^	574 (362–762)	362 (252–580)	601 (404–792)	0.001	413 (289–640)	597 (404–790)	0.022	353 (258–574)	601 (405–805)	<0.001*
Current CD4:CD8 ratio^f^	0.74 (0.50–1.08)	0.59 (0.38–0.95)	0.78 (0.58–1.12)	0.063	0.67 (0.42–0.94)	0.81 (0.56–1.13)	0.085	0.58 (0.39–0.76)	0.80 (0.57–1.13)	0.016
HIV RNA <50 copies/ml^e^	141 (94.0%)	25 (96.2%)	116 (93.5%)	1.000	40 (97.6%)	101 (92.7%)	0.445	26 (96.3%)	115 (93.5%)	1.000
Exposure to zidovudine^e^	44 (29.3%)	7 (26.9%)	37 (29.8%)	0.767	11 (26.8%)	33 (30.3%)	0.680	8 (29.6%)	36 (29.3%)	0.970
Exposure to stavudine^e^	41 (27.3%)	11 (42.3%)	30 (24.2%)	0.060	18 (43.9%)	23 (21.1%)	0.005	13 (48.1%)	28 (22.8%)	0.007
Hepatitis B infection^e^	14 (9.5%)	4 (15.4%)	10 (8.2%)	0.271	5 (12.2%)	9 (8.4%)	0.534	4 (14.8%)	10 (8.3%)	0.287
Hepatitis C infection^e^	9 (6.1%)	1 (3.8%)	8 (6.6%)	1.000	3 (7.3%)	6 (5.6%)	0.708	1 (3.7%)	8 (6.6%)	1.000
Charlson's comorbidity index^f^	2 (1–4)	4 (3–4)	2 (1–3)	<0.001	3 (2–4)	2 (1–3)	<0.001	4 (3–4)	2 (1–3)	<0.001
Multimorbidity^e^, ^g^	67 (44.7%)	18 (69.2%)	49 (39.5%)	0.006	25 (61.0%)	42 (38.5%)	0.014	18 (66.7%)	49 (39.8%)	0.011
Polypharmacy^e^, ^h^	64 (42.7%)	20 (76.9%)	44 (35.5%)	<0.001*	26 (63.4%)	38 (34.9%)	0.002	20 (74.1%)	44 (35.8%)	<0.001*
Moderate to severe depression^e^, ^i^	16 (10.7%)	1 (3.8%)	15 (12.1%)	0.308	3 (7.3%)	13 (11.9%)	0.559	1 (3.7%)	15 (12.2%)	0.306
Appendicular skeletal muscle mass (kg/m^2^)^d^	7.11 ± 1.23	5.98 ± 0.82	7.36 ± 1.17	<0.001	5.99 ± 0.78	7.55 ± 1.09	<0.001	5.97 ± 0.80	7.37 ± 1.17	<0.001
Handgrip strength (kg)^d^	29.3 ± 8.9	21.6 ± 6.3	30.9 ± 8.5	<0.001	23.5 ± 5.9	31.5 ± 8.9	<0.001	21.3 ± 4.9	31.1 ± 8.6	<0.001
6‐metre walk gait speed (m/second)^d^	1.06 ± 0.22	0.83 ± 0.19	1.11 ± 0.20	<0.001	0.90 ± 0.20	1.12 ± 0.20	<0.001	0.86 ± 0.20	1.11 ± 0.20	<0.001
Five‐time chair stand test (second)^d^	11.6 ± 4.0	14.9 ± 5.4	11.0 ± 3.4	<0.001	13.5 ± 4.8	10.9 ± 3.5	<0.001	14.6 ± 5.3	11.0 ± 3.4	<0.001
Short Physical Performance Battery^f^	11 (10–12)	9 (8–10)	12 (11–12)	<0.001	10 (8–11)	12 (11–12)	<0.001	10 (8–10.5)	12 (11–12)	<0.001

^a^ Comparison between those with and without sarcopenia according to AWGS 2014 criteria.

^b^ Comparison between those with and without sarcopenia according to AWGS 2019 criteria.

^c^ Comparison between those with and without severe sarcopenia according to AWGS 2019 criteria.

^d^ Data presented in mean ± standard deviation.

^e^ Data presented in number (percentage).

^f^ Data presented in median (25th percentile–75th percentile).

^g^ Multimorbidity is defined as the presence of two or more chronic comorbidities.

^h^ Polypharmacy is defined as taking five or more non‐anti‐retroviral drugs.

^i^ Moderate to severe depression is defined as a PHQ‐9 score of ≥10.

* *p* = 0.05 on multivariable binary logistic regression model (Details are presented in Tables S1–S3).

Abbreviation: AWGS, Asia Working Group for Sarcopenia.

**Figure 1:**
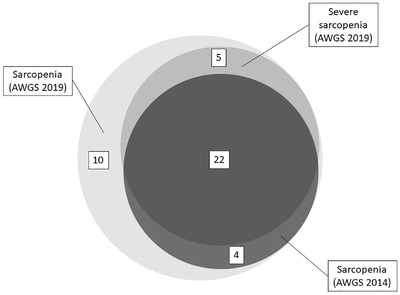
Venn diagram showing the number of participants with sarcopenia and severe sarcopenia diagnosed by AWGS‐2014 and 2019 definitions. Abbreviation: AWGS, Asia Working Group for Sarcopenia.

Among all participants, 26 (17.3%) and 41 (27.3%) had sarcopenia as defined by AWGS‐2014 and AWGS‐2019 criteria, respectively, while 27 (18.0%) had severe sarcopenia according to AWGS‐2019 criteria (Figure 1). Among the 41 participants diagnosed to have sarcopenia by AWGS‐2019 definition, all had low muscle mass, 31 (75.6%) had low muscle strength, with an additional number of 10 (24.4%) participants having low physical performance alone. Agreement between AWGS‐2014 and 2019 definitions of sarcopenia was good (kappa 0.716), and so was agreement between AWGS‐2014 definition of sarcopenia and severe sarcopenia defined by AWGS‐2019 definition (kappa 0.794).

Both definitions of sarcopenia and severe sarcopenia were associated with older age, lower level of education, lack of employment, lower CD4 count, higher Charlson's comorbidity index, multimorbidity and polypharmacy. On multivariable analyses, sarcopenia defined by AWGS‐2014 was independently associated with age, education and polypharmacy, while sarcopenia defined by AWGS‐2019 was independently associated with age (Table 1 and Tables S1–S3).

The associations between different definitions of sarcopenia and various health outcomes are presented in Table 2. Sarcopenia defined by AWGS‐2014 criteria and severe sarcopenia defined by AWGS‐2019 criteria were associated with problems with mobility, physical functioning domain and PCS of SF‐36. Both definitions of sarcopenia and severe sarcopenia were associated with problems with usual activities and impairment in IADL.

**Table 2:** Disability and quality of life measures of all participants and participants with sarcopenia

		AWGS‐2014			AWGS‐2019					
Variables	All participants *N* = 150	Sarcopenia *n* = 26	No sarcopenia *n* = 124	*p* ^a^	Sarcopenia *n* = 41	No sarcopenia *n* = 109	*p* ^b^	Severe sarcopenia *n* = 27	No severe sarcopenia *n* = 123	*p* ^c^
EQ‐5D‐5L index^d^	0.87 ± 0.17	0.83 ± 0.15	0.88 ± 0.17	0.208	0.83 ± 0.19	0.88 ± 0.16	0.092	0.83 ± 0.15	0.88 ± 0.17	0.196
EQ‐VAS^d^	76.9 ± 14.24	75.7 ± 14.8	77.1 ± 14.2	0.649	75.9 ± 15.4	77.2 ± 13.8	0.627	74.7 ± 15.5	77.3 ± 14.0	0.388
EQ‐5D‐5L domains										
Problem with mobility^e^	20 (13.3%)	9 (34.6%)	11 (8.9%)	0.002	9 (22.0%)	11 (10.1%)	0.057	9 (33.3%)	11 (8.9%)	0.002
Problem with self‐care^e^	7 (4.7%)	1 (3.8%)	6 (4.8%)	1.000	3 (7.3%)	4 (3.7%)	0.392	2 (7.4%)	5 (4.1%)	0.610
Problem with usual activities^e^	18 (12.0%)	8 (30.8%)	10 (8.1%)	0.004	10 (24.4%)	8 (7.3%)	0.009	8 (29.6%)	10 (8.1%)	0.005
Pain or discomfort^e^	81 (54.0%)	18 (69.2%)	63 (50.8%)	0.087	26 (63.4%)	55 (50.5%)	0.156	17 (63.0%)	64 (52.0%)	0.302
Anxiety or depression^e^	59 (39.3%)	9 (34.6%)	50 (40.3%)	0.588	17 (41.5%)	42 (38.5%)	0.743	11 (40.7%)	48 (39.0%)	0.869
SF‐36 domain scores										
Physical functioning^d^	84.2 ± 17.2	77.3 ± 18.7	85.7 ± 16.6	0.024	80.5 ± 20.0	85.6 ± 15.9	0.105	77.0 ± 18.0	85.8 ± 16.7	0.016
Role limitations due to physical health^d^	74.8 ± 38.6	63.5 ± 44.3	77.2 ± 37.0	0.149	67.1 ± 42.7	77.8 ± 36.7	0.131	63.9 ± 45.1	77.2 ± 36.8	0.160
Role limitations due to emotional problems^d^	74.9 ± 39.4	67.9 ± 45.7	76.3 ± 38.0	0.388	71.5 ± 42.5	76.1 ± 38.2	0.525	74.1 ± 41.7	75.1 ± 39.0	0.906
Vitality^d^	64.8 ± 19.4	63.3 ± 20.8	65.1 ± 19.2	0.660	64.6 ± 21.0	64.9 ± 18.9	0.949	64.6 ± 20.6	64.8 ± 19.3	0.960
Emotional wellbeing^d^	71.8 ± 18.6	72.2 ± 19.5	71.7 ± 18.5	0.906	71.0 ± 20.0	72.0 ± 18.1	0.767	73.5 ± 18.9	71.4 ± 18.6	0.597
Social functioning^d^	83.8 ± 21.1	84.6 ± 20.4	83.7 ± 21.3	0.836	83.8 ± 22.9	71.0 ± 20.0	0.998	85.6 ± 20.1	83.4 ± 21.4	0.623
Bodily pain^d^	75.4 ± 23.2	69.5 ± 24.2	76.6 ± 22.9	0.159	70.1 ± 26.0	77.3 ± 21.8	0.087	71.8 ± 25.2	76.1 ± 22.8	0.376
General health^d^	53.7 ± 18.3	52.1 ± 17.9	54.0 ± 18.4	0.628	53.7 ± 17.4	53.7 ± 18.7	0.986	52.8 ± 17.8	53.9 ± 18.4	0.773
Physical component score^d^	45.2 ± 10.9	40.8 ± 12.0	46.1 ± 10.5	0.022	42.5 ± 12.4	46.2 ± 10.2	0.066	40.7 ± 11.9	46.1 ± 10.5	0.019
Mental component score^d^	51.0 ± 12.1	51.7 ± 11.5	50.9 ± 12.2	0.747	51.3 ± 11.8	50.9 ± 12.2	0.873	53.1 ± 10.7	50.6 ± 12.3	0.327
Disability										
Impaired ADL^e^	3 (2.0%)	1 (3.8%)	2 (1.6%)	0.437	1 (2.4%)	2 (1.8%)	1.000	1 (3.7%)	2 (1.6%)	0.451
Impaired IADL^e^	16 (10.7%)	7 (26.9%)	9 (7.3%)	0.008	9 (22.0%)	7 (6.4%)	0.014	6 (22.2%)	10 (8.1%)	0.043

^a^ Comparison between those with and without sarcopenia according to AWGS 2014 criteria.

^b^ Comparison between those with and without sarcopenia according to AWGS 2019 criteria.

^c^ Comparison between those with and without severe sarcopenia according to AWGS 2019 criteria.

^d^ Data presented in mean ± standard deviation.

^e^ Data presented in number (percentage).

Abbreviation: AWGS, Asia Working Group for Sarcopenia.

As for the individual components of the definitions of sarcopenia, muscle mass was not associated with any of the health outcomes, while handgrip strength, five‐time chair stand test, gait speed and SPPB score were associated with one or more of the health outcomes, including EQ‐5D‐5L index, several EQ‐5D‐5L domains, physical functioning and PCS of SF‐36, and impaired ADL and IADL (Table S4). Moreover, the different cut‐offs for handgrip strength, gait speed and criteria for low physical performance in the AWGS‐2014 and AWGS‐2019 definitions had similar correlations with these health outcome measures (Tables S5a and 5b).

One or more of the definitions of sarcopenia were associated with a problem with mobility, a problem with usual activities, physical functioning, PCS and impairment in IADL, in the unadjusted and/or adjusted models. The other health outcomes did not show a significant correlation with any of the sarcopenia definitions. Table 3 shows the significant associations between various definitions of sarcopenia and health outcomes. Both sarcopenia defined by AWGS‐2014 and severe sarcopenia retained an association with a problem with mobility and usual activities, impairment in IADL and PCS, after adjusting for age and sex. Both definitions of sarcopenia and severe sarcopenia were independently associated with impairment in IADL in the fully adjusted models. Sarcopenia defined by AWGS‐2019 had a trend of association with the EQ‐5D‐5L index and PCS of SF‐36.

**Table 3:** Association between sarcopenia by different criteria and selected health outcomes^a^, ^b^

	AWGS 2014			AWGS 2019			Severe sarcopenia		
Health outcomes	Unadjusted odds ratio (95% confidence interval), *p‐*value	Model 1^c^	Model 2^d^	Unadjusted odds ratio (95% confidence interval), *p‐*value	Model 1^c^	Model 2^d^	Unadjusted odds ratio (95% confidence interval), *p‐*value	Model 1^c^	Model 2^d^
Problem with mobility	**5.439 (1.965**–**15.050), *p* = 0.001**	**5.439 (1.965**–**15.050), *p* = 0.001**	2.948 (0.943–9.212), *p* = 0.063	2.506 (0.953–6.591), *p* = 0.063	1.618 (0.552–4.740), *p* = 0.380	1.618 (0.552–4.740), *p* = 0.380	**5.091 (1.851**–**14.003), *p* = 0.002**	**5.091 (1.851**–**14.003), *p* = 0.002**	**3.645 (1.256**–**10.576), *p* = 0.017**
Problem with usual activities	**5.067 (1.766**–**14.540), *p* = 0.003**	**5.067 (1.766**–**14.540), *p* = 0.003**	**3.178 (1.040**–**9.708), *p* = 0.042**	**4.073 (1.479**–**11.216), *p* = 0.007**	**4.073 (1.479**–**11.216), *p* = 0.007**	**4.073 (1.479**–**11.216), *p* = 0.007**	**4.758 (1.667**–**13.583), *p* = 0.004**	**4.758 (1.667**–**13.583), *p* = 0.004**	2.004 (0.630–6.369), *p* = 0.239
Impaired IADL	**4.708 (1.566**–**14.150), *p* = 0.006**	**5.237 (1.697**–**16.161), *p* = 0.004**	**5.237 (1.697**–**16.161), *p* = 0.004**	**4.098 (1.413**–**11.883), *p* = 0.009**	**4.451 (1.512**–**13.103), *p* = 0.007**	**4.451 (1.512**–**13.103), *p* = 0.007**	**3.229 (1.060**–**9.837), *p* = 0.039**	**3.494 (1.123**–**10.875), *p* = 0.031**	**3.494 (1.123**–**10.875), *p* = 0.031**
Health outcomes	Unadjusted unstandardized beta coefficient, *p*‐value	Model 1^e^	Model 2^f^	Unadjusted unstandardized beta coefficient, *p*‐value	Model 1^e^	Model 2^f^	Unadjusted unstandardized beta coefficient, *p*‐value	Model 1^e^	Model 2^f^
EQ‐5D‐5L index	–0.046, *p* = 0.208	–0.046, *p* = 0.208	–0.023, *p* = 0.586	–0.052, *p* = 0.092	–0.052, *p* = 0.092	–0.052, *p* = 0.092	–0.046, *p* = 0.196	–0.046, *p* = 0.196	–0.032, *p* = 0.428
Physical functioning	–**8.337, *p* = 0.024**	–4.719, *p* = 0.243	0.673, *p* = 0.869	–5.109, *p* = 0.105	–1.856, *p* = 0.583	–1.856, *p* = 0.583	–**8.735, *p* = 0.016**	–5.181, *p* = 0.197	–2.040, *p* = 0.613
Physical component score	–**5.342, *p* = 0.022**	–**5.342, *p* = 0.022**	–1.305, *p* = 0.618	–3.661, *p* = 0.066	–3.661, *p* = 0.066	–3.661, *p* = 0.066	–**5.412, *p* = 0.019**	–**5.412, *p* = 0.019**	–2.287, *p* = 0.346

^a^ Health outcomes with significant association with one or more definitions of sarcopenia on univariate analysis are presented in this table.

^b^ Associations with *p*‐value < 0.05 were presented in bold type.

^c^ Model 1 shows the odds ratio, adjusted for age and sex.

^d^ Model 2 shows the odds ratio adjusted for age, sex and all variables with significant independent association with sarcopenia.

^e^ Model 1 shows the beta coefficient, adjusted for age and sex.

^f^ Model 2 shows an unstandardized beta coefficient, adjusted for age, sex and all variables with significant independent association with sarcopenia.

Abbreviation: AWGS, Asia Working Group for Sarcopenia.

## 4 DISCUSSION

In this study, we aimed to determine the prevalence of sarcopenia using the two AWGS definitions and whether health outcomes were associated with these definitions of sarcopenia among Asian PLWH. The prevalence of sarcopenia defined by AWGS‐2014 and AWGS‐2019 and severe sarcopenia was 17.3%, 27.3% and 18.0%, respectively. Different definitions of sarcopenia were independently associated with age, education level, current CD4 count and/or polypharmacy. The AWGS‐2014 definition of sarcopenia and severe sarcopenia defined by AWGS‐2019 was associated with more health‐related QOL outcome measures, while both definitions of sarcopenia and severe sarcopenia were associated with functional disability.

A recent meta‐analysis revealed that PLWH had a 13% pooled prevalence of sarcopenia when using both muscle mass and muscle function to define sarcopenia [6]. Few studies have been performed in Asian populations to evaluate the prevalence of sarcopenia among PLWH [6]. Two studies performed in India defined sarcopenia using low muscle mass alone showing a prevalence of 40% in middle‐aged men [21] and 18% in pre‐menopausal women [22]. In one study performed in Malaysia, involving predominantly Chinese PLWH, the prevalence of sarcopenia (defined by AWGS‐2014 criteria) was 8%, while the prevalence in a subgroup of PLWH older than 50 years was 17% [8].

Studies performed in the general Asian older adult populations with a large sample size revealed a consistent prevalence of sarcopenia ranging from 6% to 12% using the AWGS‐2014 criteria [13, 23], while more recent studies using AWGS‐2019 criteria demonstrated the prevalence of sarcopenia ranging from 11% to 18% [24, 25]. Our findings supported previous evidence showing a higher prevalence of sarcopenia among PLWH compared with matched HIV‐uninfected populations [6, 8, 21, 22, 26].

We have identified older age, lower education level, lower current CD4 count and presence of polypharmacy as risk factors associated with sarcopenia. Older age, lower education level and lower CD4 count have previously been shown to correlate with sarcopenia or low muscle mass [6, 8, 27, 28]. On the other hand, the correlation between polypharmacy and sarcopenia was less well studied among PLWH. In a recent scoping review evaluating the relationship between sarcopenia and polypharmacy in the general population, polypharmacy and the number of medications were independently associated with sarcopenia in community‐dwelling older adults in the majority of studies [29]. One longitudinal study in Japan demonstrated polypharmacy as an independent predictor of new‐onset sarcopenia over a 5‐year follow‐up period [30]. Possible explanations include direct muscle toxicities via mitochondrial and metabolic pathways of multiple classes of medications and indirect mechanisms involving poor nutrition and reduced physical activity due to polypharmacy [31]. Moreover, the rate of polypharmacy is higher in those with comorbidity [32, 33]. Polypharmacy can be an indirect marker of increased comorbidity, which is also a risk factor for sarcopenia.

We observed a higher prevalence of sarcopenia when the AWGS‐2019 definition was adopted, as compared with the AWGS‐2014 definition. Similar observations were made in non‐HIV‐infected populations in Asia [24]. AWGS‐2019 definition has more lenient cut‐offs for defining weak handgrip strength and slow gait speed, and includes additional criteria of chair stand test and SPPB in defining physical performance. These changes resulted in more PLWH fulfilling the diagnosis of sarcopenia under the AWGS‐2019 definition.

With the changes in the criteria defining sarcopenia, it would be important to determine which is the more appropriate definition to be adopted in PLWH [20]. To evaluate this issue, we determined the correlation between different definitions of sarcopenia and health outcomes, including disability and QOL.

EQ‐5D‐5L and SF‐36 were both commonly used instruments for the assessment of QOL among individuals with sarcopenia in the general population. For example, in determining the construct validity of a sarcopenia‐specific QOL measure, it was shown to have a positive correlation with both the EQ‐5D‐5L index as well as individual domain scores of these tools, including physical functioning of SF‐36 and the domains of mobility and usual activities in EQ‐5D‐5L [34]. Therefore, these measures of both collective scores and individual domains will be useful in reflecting QOL among people with sarcopenia. In general populations of older adults, sarcopenia was associated with reduced health‐related QOL, as measured by EQ‐5D‐5L index [35, 36, 37] and domains of mobility, self‐care, usual activity [35, 36, 37] and physical functioning [38, 39, 40]. The findings of our study population of PLWH were consistent with these observations.

The AWGS‐2014 sarcopenia definition and severe sarcopenia in AWGS‐2019 identified individuals with the most severe loss of muscle mass and function. Therefore, not unexpectedly, participants with sarcopenia in these two categories in our study had the most correlation with parameters associated with worse physical health‐related QOL. In other words, these two definitions were able to identify individuals with the highest risk of poor health outcomes, and evidence‐based interventions for sarcopenia to prevent future disability are urgently required for these individuals [41].

On the other hand, it is uncertain whether the less stringent AWGS‐2019 definition of sarcopenia would include individuals who are in fact not at risk for poor health outcomes. We observed that the AWGS‐2019 definition correlated with impairment in IADL and had a trend of correlation with poorer health‐related QOL. The lower thresholds of handgrip strength, gait speed and physical performance in AWGS‐2019 also had similar correlations with health outcome measures as the higher thresholds adopted in AWGS‐2014 (Tables S5a and 5b). This suggested that these revised criteria for defining sarcopenia can potentially contribute to the early identification of PLWH at risk of functional disability, without over‐diagnosing sarcopenia in low‐risk individuals. These changes in AWGS sarcopenia definition can foster sarcopenia prevention and treatment programmes among PLWH. Implementing interventions at an early stage for the treatment of sarcopenia can potentially improve their QOL and avoid the development of disability in the future. We suggest that future research involving longitudinal study design and a larger sample size should be conducted to evaluate the correlation of incidence of poor health outcomes, including functional disability, hospitalization and mortality, with the revised definition of sarcopenia among Asian populations of PLWH.

The increase in the prevalence of sarcopenia with the changes in AWGS definitions was in contrast to a reduction in the prevalence of sarcopenia among PLWH with the change from EWGSOP1 to EWGSOP2 [20]. This reduction was partly due to more conservative cut‐offs for the determination of low muscle strength and low muscle mass in EWGSOP2. Moreover, in EWGSOP2, physical performance was only adopted to define severe sarcopenia but not sarcopenia, while AWGS‐2019 expanded the measures for physical performance to define sarcopenia. There were concerns with these changes in the revised EWGSOP2 criteria, which would limit its use in identifying at‐risk PLWH with sarcopenia and prevent the implementation of early interventions for the avoidance of progression to disability [20, 42]. Our findings supported both AWGS and EWGSOP guidelines in including both muscle mass and muscle strength in defining sarcopenia. While muscle mass was not associated with any of the health outcome measures, muscle strength, as represented by handgrip strength and/or five‐time chair stand test, correlated with several measures of QOL and disability. On the other hand, measures of physical performance, including gait speed and SPPB, correlated with even more health outcome measures (Table S4). These parameters had also been shown to predict mortality among PLWH [43, 44]. Our findings, therefore, supported AWGS‐2019 in including the presence of either muscle strength or physical performance in the definition of sarcopenia. Monitoring of these parameters in older PLWH is important in the clinical setting to identify individuals who would benefit from interventions to improve physical function.

There are some limitations to our study. Firstly, we included a relatively small sample size involving PLWH of Chinese ethnicity. The results may not be generalizable to other Asian populations of PLWH. Secondly, this was a cross‐sectional study, thus we could not make conclusions around the causation of the risk factors associated with sarcopenia. Likewise, a longitudinal study in the future will be needed to determine whether an early diagnosis of sarcopenia with the AWGS‐2019 definition can be adopted to predict incident disability and other health outcomes. Longitudinal studies would also allow the determination of the impact of different interventions in treating sarcopenia on a decline in physical function and development of disability. Thirdly, the thresholds used to define low muscle mass, weak handgrip strength, slow gait speed and low physical performance were derived from older HIV‐uninfected adults for AWGS definition. It is likely that this may under‐estimate the true prevalence of sarcopenia among our cohort of PLWH, who had a younger chronological age. Future studies involving age‐ and sex‐matched HIV‐uninfected controls to determine the prevalence of sarcopenia among PLWH would be important. Moreover, we did not include a gold standard for measuring muscle mass (e.g. computed tomography scan [45]), this limited our determination of sensitivity and specificity of each definition of sarcopenia against a reference standard.

## 5 CONCLUSIONS

Among this sample of Chinese PLWH, the AWGS‐2019 definition identified a higher prevalence of sarcopenia compared with the AWGS‐2014 definition. While the AWGS‐2014 definition and severe sarcopenia defined by the AWGS‐2019 criteria correlated with the most parameters of poorer health‐related QOL, the AWGS‐2019 definition could potentially identify early‐risk individuals who may benefit from early interventions to prevent future disability.

## COMPETING INTERESTS

GL has received research grants and consultancy fees from Gilead Sciences, MSD and ViiV.

## AUTHORS’ CONTRIBUTIONS

FWLL: data collection, data analysis, writing original draft, review and edit writing. TL, HYH and YYC: data collection and review writing. SKC and VW: data collection, data analysis and review writing. TCYK: conceptualization, methodology and review writing. GL: conceptualization, methodology, data collection, data analysis, writing original draft, review and edit writing, supervision. All authors have read and approved the final manuscript.

## FUNDING

This study was supported by the Hong Kong AIDS Trust Fund (MSS 319R).

## Supporting information


**Table S1**. Multivariate regression analyses of variables associated with AWGS 2014 definition of sarcopenia.
**Table S2**. Multivariate regression analyses of variables associated with AWGS 2019 definition of sarcopenia.
**Table S3**. Multivariate regression analyses of variables associated with severe sarcopenia (AWGS 2019).
**Table S4**. Correlations between muscle mass, muscle strength and physical performance parameters and health‐related quality of life and disability.
**Table S5a**. Correlations between different cutoffs of muscle strength (handgrip strength) and gait speed and health‐related quality of life and disability.
**Table S5b**. Correlations between different cutoffs of five chair stand test, short physical performance battery (SPPB) and physical performance parameters and health‐related quality of life and disability.

## Data Availability

The data used or analysed during this study are available from the corresponding author on reasonable request.
