# Supplementary material for: Sarcopenia in people living with HIV in Hong Kong: which definition correlates with health outcomes?
Source: J Int AIDS Soc. 2022 Sep 29;25(Suppl 4):e25988. doi: 10.1002/jia2.25988 (PMC9522638; doi:10.1002/jia2.25988)
Supplement: Supplementary file 1 — Table S1. Multivariate regression analyses of variables associated with AWGS 2014 definition of sarcopenia. Table S2. Multivariate regression analyses of variables associated with AWGS 2019 definition of sarcopenia. Table S3. Multivariate regression analyses of variables associated with severe sarcopenia (AWGS 2019). Table S4. Correlations between muscle mass, muscle strength and physical performance parameters and health‐related quality of life and disability. Table S5a. Correlations between different cutoffs of muscle strength (handgrip strength) and gait speed and health‐related quality of life and disability. Table S5b. Correlations between different cutoffs of five chair stand test, short physical performance battery (SPPB) and physical performance parameters and health‐related quality of life and disability. [file JIA2-25-e25988-s001.docx]

**Supplementary Table 1. Multivariate regression analyses of variables associated with AWGS 2014 definition of sarcopenia**

| Variables | Adjusted odds ratio | 95% confidence interval | p | Variance inflation factor |
| --- | --- | --- | --- | --- |
| Age | 1.11 | 1.04-1.20 | 0.003 | 1.879 |
| Male | 2.69 | 0.58-12.5 | 0.207 | 1.075 |
| Secondary education or above | 3.68 | 1.22-11.09 | 0.021 | 1.219 |
| No employment | 0.57 | 0.14-2.32 | 0.431 | 1.542 |
| Current CD4 count | 0.998 | 0.995-1.000 | 0.070 | 1.160 |
| Multimorbidity | 1.26 | 0.35-4.49 | 0.722 | 1.339 |
| Polypharmacy | 4.05 | 1.28-12.8 | 0.017 | 1.369 |

**Supplementary Table 2. Multivariate regression analyses of variables associated with AWGS 2019 definition of sarcopenia**

| Variables | Adjusted odds ratio | 95% confidence interval | p | Variance inflation factor |
| --- | --- | --- | --- | --- |
| Age | 1.11 | 1.06-1.17 | <0.001 | 1.963 |
| Male | 1.73 | 0.51-5.84 | 0.376 | 1.084 |
| Secondary education or above | 1.72 | 0.65-4.57 | 0.273 | 1.226 |
| No employment | 0.82 | 0.29-2.33 | 0.711 | 1.563 |
| Duration of HIV diagnosis | 1.03 | 0.96-1.09 | 0.426 | 1.267 |
| Current CD4 count | 0.999 | 0.998-1.001 | 0.439 | 1.247 |
| Exposure of stavudine | 2.21 | 0.95-5.16 | 0.067 | 1.186 |
| Multimorbidity | 1.14 | 0.44-2.96 | 0.790 | 1.415 |
| Polypharmacy | 1.95 | 0.85-4.48 | 0.116 | 1.405 |

**Supplementary Table 3. Multivariate regression analyses of variables associated with severe sarcopenia (AWGS 2019)**

| Variables | Adjusted odds ratio | 95% confidence interval | p | Variance inflation factor |
| --- | --- | --- | --- | --- |
| Age | 1.14 | 1.07-1.22 | <0.001 | 1.948 |
| Male | 2.54 | 0.56-11.50 | 0.226 | 1.085 |
| Secondary education or above | 2.42 | 0.81-7.29 | 0.116 | 1.229 |
| No employment | 0.67 | 0.17-2.71 | 0.580 | 1.567 |
| Current CD4 count | 0.997 | 0.995-1.000 | 0.034 | 1.817 |
| Current CD4:CD8 ratio | 0.56 | 0.11-2.76 | 0.477 | 1.630 |
| Exposure of stavudine | 1.78 | 0.58-5.46 | 0.315 | 1.133 |
| Multimorbidity | 1.09 | 0.29-4.06 | 0.894 | 1.406 |
| Polypharmacy | 3.85 | 1.28-11.55 | 0.016 | 1.371 |

**Supplementary Table 4. Correlations between muscle mass, muscle strength and physical performance parameters and health-related quality of life and disability**

|  | **Height-adjusted Muscle mass** | **p** | **Handgrip strength** | **p** | **Five chair stand test** | **p** | **Gait speed** | **p** | **SPPB** | **p** |
| --- | --- | --- | --- | --- | --- | --- | --- | --- | --- | --- |
| **EQ-5D-5L index^1^** | -0.001 | 0.947 | **0.004** | **0.013** | **-0.012** | **<0.001** | **0.192** | **0.002** | **0.028** | **0.001** |
| **EQ-VAS^1^** | 1.503 | 0.121 | **0.331** | **0.011** | -0.158 | 0.580 | **15.612** | **0.003** | 1.268 | 0.070 |
| **EQ-5D-5L domains** | | | | | | | | | | |
| Problem with mobility^2^ | -0.138 | 0.488 | **-0.085** | **0.004** | **0.329** | **<0.001** | **-7.550** | **<0.001** | **-0.660** | **<0.001** |
| Problem with self-care^2^ | 0.048 | 0.879 | **-0.108** | **0.023** | **0.224** | **0.004** | **-3.673** | **0.023** | **-0.408** | **0.019** |
| Problem with usual activities^2^ | -0.308 | 0.150 | **-0.157** | **<0.001** | **0.311** | **<0.001** | **-5.947** | **<0.001** | **-0.571** | **<0.001** |
| Pain or discomfort^2^ | 0.031 | 0.819 | -0.016 | 0.399 | 0.066 | 0.150 | **-1.653** | **0.035** | **-0.249** | **0.025** |
| Anxiety or depression^2^ | 0.040 | 0.775 | -0.017 | 0.357 | 0.002 | 0.960 | 0.733 | 0.338 | 0.057 | 0.579 |
| **SF-36 domain scores** | | | | | | | | | | |
| Physical functioning^1^ | 1.794 | 0.128 | **0.628** | **<0.001** | **-2.038** | **<0.001** | **38.797** | **<0.001** | **4.976** | **<0.001** |
| Role limitations due to physical health^1^ | 1.290 | 0.626 | 0.489 | 0.169 | -1.434 | 0.063 | **42.148** | **0.003** | **4.835** | **0.010** |
| Role limitations due to emotional problems^1^ | 0.216 | 0.937 | 0.193 | 0.595 | -0.616 | 0.448 | 27.029 | 0.061 | 1.323 | 0.497 |
| Vitality^1^ | 0.107 | 0.936 | 0.240 | 0.179 | -0.585 | 0.138 | 14.921 | 0.036 | 1.387 | 0.148 |
| Emotional well-being^1^ | 0.468 | 0.716 | 0.097 | 0.572 | **-0.773** | **0.041** | 6.429 | 0.348 | 0.820 | 0.372 |
| Social functioning^1^ | -1.989 | 0.168 | -0.122 | 0.531 | **-0.858** | **0.041** | 5.000 | 0.520 | 1.112 | 0.286 |
| Bodily pain^1^ | 0.389 | 0.806 | 0.246 | 0.250 | -0.539 | 0.244 | **19.338** | **0.022** | **1.770** | **0.122** |
| General health^1^ | 0.114 | 0.928 | 0.314 | 0.061 | -0.610 | 0.097 | **18.555** | **0.005** | **2.184** | **0.015** |
| Physical component score^1^ | 0.888 | 0.236 | **0.352** | **<0.001** | **-0.924** | **<0.001** | **21.920** | **<0.001** | **2.692** | **<0.001** |
| Mental component score^1^ | -0.572 | 0.491 | -0.073 | 0.515 | -0.122 | 0.625 | -0.211 | 0.962 | -0.223 | 0.710 |
| **Disability** | | | | | | | | | | |
| Impaired ADL^2^ | 0.128 | 0.784 | -0.111 | 0.111 | **0.204** | **0.008** | **-5.115** | **0.032** | -0.466 | 0.054 |
| Impaired IADL^2^ | -0.084 | 0.698 | **-0.084** | **0.010** | **0.297** | **0.001** | **-4.332** | **0.001** | **-0.527** | **<0.001** |

^1^ B coefficients for linear regression analyses are shown in these rows. ^2^ B coefficients for logistic regression analyses are shown in these rows. Correlations with p value <0.05 are shown in bold type.

**Supplementary Table 5a. Correlations between different cutoffs of muscle strength (handgrip strength) and gait speed and health-related quality of life and disability**

|  | AWGS 2014 | | | AWGS 2019 | | | AWGS 2014 | | | AWGS 2019 | | |
| --- | --- | --- | --- | --- | --- | --- | --- | --- | --- | --- | --- | --- |
| Variables | Normal handgrip strength  N=109 | Weak handgrip strength  N=41 | p | Normal handgrip strength  N=98 | Weak handgrip strength  N=52 | p | Normal gait speed  N=136 | Slow gait speed  N=14 | p | Normal gait speed  N=98 | Slow gait speed  N=52 | p |
| **EQ-5D-5L index** | 0.89±0.15 | 0.82±0.20 | 0.053 | **0.89±0.15** | **0.82±0.19** | **0.016** | **0.88±0.16** | **0.78±0.19** | **0.049** | **0.89±0.15** | **0.82±0.18** | **0.019** |
| **EQ-VAS** | **78.6±13.4** | **72.2±15.6** | **0.022** | **78.8±13.6** | **73.1±14.8** | **0.019** | 77.5±14.2 | 70.9±13.1 | 0.102 | 78.6±13.1 | 73.6±15.8 | 0.058 |
| **EQ-5D-5L domains** | | | | | | | | | | | | |
| Problem with mobility | **9 (8.3%)** | **11 (26.8%)** | **0.003** | **9 (9.2%)** | **11 (21.2%)** | **0.047** | **13 (9.6%)** | **7 (50.0%)** | **0.001** | **2 (2.0%)** | **18 (34.6%)** | **<0.001** |
| Problem with self-care | 3 (2.8%) | 4 (9.8%) | 0.070 | **2 (2.0%)** | **5 (9.6%)** | **0.049** | 5 (3.7%) | 2 (14.3%) | 0.130 | **2 (2.0%)** | **5 (9.6%)** | **0.049** |
| Problem with usual activities | **6 (5.5%)** | **12 (29.3%)** | **<0.001** | **6 (6.1%)** | **12 (23.1%)** | **0.002** | **12 (8.8%)** | **6 (42.9%)** | **0.002** | **3 (3.1%)** | **15 (28.8%)** | **<0.001** |
| Pain or discomfort | 56 (51.4%) | 25 (61.0%) | 0.293 | 48 (49.0%) | 33 (63.5%) | 0.090 | 71 (52.2%) | 10 (71.4%) | 0.169 | 49 (50.0%) | 32 (61.5%) | 0.177 |
| Anxiety or depression | 43 (39.4%) | 16 (39.0%) | 0.962 | 35 (35.7%) | 24 (46.2%) | 0.213 | 56 (41.2%) | 3 (21.4%) | 0.150 | 42 (42.9%) | 17 (32.7%) | 0.225 |
| **SF-36 domain scores** | | | | | | | | | | | | |
| Physical functioning | **88.1±14.5** | **73.9±19.7** | **<0.001** | **87.9±14.9** | **77.2±19.1** | **<0.001** | **86.0±15.0** | **66.4±26.3** | **0.016** | **90.0±10.3** | **73.3±21.8** | **<0.001** |
| Role limitations due to physical health | 78.2±35.5 | 65.9±45.0 | 0.119 | 78.8±35.1 | 67.3±43.9 | 0.105 | 77.2±36.6 | 51.8±50.4 | 0.087 | **81.9±33.1** | **61.5±44.7** | **0.005** |
| Role limitations due to emotional problems | 74.9±38.3 | 74.8±42.7 | 0.986 | 75.2±38.1 | 74.4±42.1 | 0.905 | 75.2±38.7 | 71.4±46.9 | 0.731 | 78.9±35.9 | 67.3±44.5 | 0.109 |
| Vitality | 65.8±18.8 | 62.2±21.0 | 0.316 | 65.3±18.8 | 64.0±20.8 | 0.695 | 65.2±19.5 | 61.1±18.7 | 0.453 | 67.1±19.2 | 60.5±19.4 | 0.049 |
| Emotional well-being | 71.9±17.9 | 71.5±20.6 | 0.921 | 71.7±17.6 | 71.9±20.6 | 0.967 | 72.0±18.5 | 69.1±19.7 | 0.582 | 72.3±18.2 | 70.8±19.4 | 0.636 |
| Social functioning | 84.1±20.4 | 83.2±23.2 | 0.831 | 84.3±20.1 | 82.9±23.1 | 0.705 | 83.8±21.4 | 83.9±19.3 | 0.986 | 84.4±21.1 | 82.7±21.3 | 0.631 |
| Bodily pain | 77.5±22.3 | 69.6±24.8 | 0.061 | 78.0±22.0 | 70.3±24.6 | 0.051 | 76.2±23.3 | 67.1±20.8 | 0.165 | **78.9±20.9** | **68.6±25.9** | **0.009** |
| General health | **56.3±17.2** | **46.8±19.5** | **0.004** | 55.6±17.6 | 50.2±19.1 | 0.087 | 54.6±18.4 | 45.0±15.2 | 0.061 | 55.7±18.7 | 49.9±17.0 | 0.063 |
| Physical component score | **47.6±9.3** | **38.7±12.1** | **<0.001** | **47.6±9.5** | **40.7±11.9** | **<0.001** | **46.3±9.6** | **34.3±16.4** | **0.018** | **48.5±7.7** | **38.8±13.1** | **<0.001** |
| Mental component score | 50.4±11.7 | 52.7±13.0 | 0.303 | 50.4±11.5 | 52.2±13.2 | 0.388 | 50.8±12.2 | 53.1±10.5 | 0.501 | 50.8±12.2 | 51.3±11.9 | 0.815 |
| **Disability** | | | | | | | | | | | | |
| Impaired ADL | **0 (0%)** | **3 (7.3%)** | **0.019** | **0 (0%)** | **3 (5.8%)** | **0.040** | 2 (1.5%) | 1 (7.1%) | 0.256 | 1 (1.0%) | 2 (3.8%) | 0.276 |
| Impaired IADL | **6 (5.5%)** | **10 (24.4%)** | **0.002** | **4 (4.1%)** | **12 (23.1%)** | **<0.001** | **10 (7.4%)** | **6 (42.9%)** | **0.001** | **4 (4.1%)** | **12 (23.1%)** | **<0.001** |

**Supplementary Table 5b. Correlations between different cutoffs of five chair stand test, short physical performance battery (SPPB) and physical performance parameters and health-related quality of life and disability**

|  | AWGS 2019 | | | AWGS 2019 | | | AWGS 2014 | | | AWGS 2019 | | |
| --- | --- | --- | --- | --- | --- | --- | --- | --- | --- | --- | --- | --- |
| Variables | Normal five chair stand test  N=95 | Slow five chair stand test  N=55 | p | Normal SPPB score  N=122 | Low SPPB score  N=28 | p | Normal physical performance  N=136 | Low physical performance  N=14 | p | Normal physical performance  N=76 | Low physical performance  N=74 | p |
| **EQ-5D-5L index** | 0.89±0.16 | 0.84±0.18 | 0.085 | **0.89±0.15** | **0.75±0.21** | **0.002** | **0.88±0.16** | **0.78±0.19** | **0.049** | 0.89±0.17 | 0.85±0.17 | 0.093 |
| **EQ-VAS** | 77.9±12.7 | 75.0±16.6 | 0.266 | 77.8±13.4 | 73.0±17.3 | 0.179 | 77.5±14.2 | 70.9±13.1 | 0.102 | 78.312.9 | 75.3±15.5 | 0.200 |
| **EQ-5D-5L domains** | | | | | | | | | | | | |
| Problem with mobility | **6 (6.3%)** | **14 (25.5%)** | **0.001** | **8 (6.6%)** | **12 (42.9%)** | **<0.001** | **13 (9.6%)** | **7 (50.0%)** | **0.001** | **1 (1.3%)** | **19 (25.7%)** | **<0.001** |
| Problem with self-care | 3 (3.2%) | 4 (7.3%) | 0.261 | **3 (2.5%)** | **4 (14.3%)** | **0.023** | 5 (3.7%) | 2 (14.3%) | 0.130 | 2 (2.6%) | 5 (6.8%) | 0.273 |
| Problem with usual activities | **5 (5.3%)** | **13 (23.6%)** | **0.001** | **7 (5.7%)** | **11 (39.3%)** | **<0.001** | **12 (8.8%)** | **6 (42.9%)** | **0.002** | **2 (2.6%)** | **16 (21.6%)** | **<0.001** |
| Pain or discomfort | 48 (50.5%) | 33 (60.0%) | 0.262 | **60 (49.2%)** | **21 (75.0%)** | **0.013** | 71 (52.2%) | 10 (71.4%) | 0.169 | 36 (47.4%) | 45 (60.8%) | 0.099 |
| Anxiety or depression | 39 (41.1%) | 20 (36.4%) | 0.571 | 48 (39.3%) | 11 (39.3%) | 0.995 | 56 (41.2%) | 3 (21.4%) | 0.150 | 31 (40.8%) | 28 (37.8%) | 0.711 |
| **SF-36 domain scores** | | | | | | | | | | | | |
| Physical functioning | **89.2±11.7** | **75.6±21.4** | **<0.001** | **88.1±11.7** | **67.1±25.4** | **<0.001** | **86.0±15.0** | **66.4±26.3** | **0.016** | **91.1±9.6** | **77.2±20.2** | **<0.001** |
| Role limitations due to physical health | 79.0±36.0 | 67.7±42.1 | 0.101 | **78.9±35.4** | **57.1±47.1** | **0.028** | 77.2±36.6 | 51.8±50.4 | 0.087 | **82.9±32.7** | **66.6±42.5** | **0.009** |
| Role limitations due to emotional problems | 76.1±36.6 | 72.7±44.0 | 0.628 | 76.5±37.8 | 67.9±45.8 | 0.359 | 75.2±38.7 | 71.4±46.9 | 0.731 | 88.7±33.7 | 68.9±43.9 | 0.068 |
| Vitality | 65.7±20.2 | 63.3±18.0 | 0.466 | **66.3±19.2** | **58.2±19.3** | **0.046** | 65.2±19.5 | 61.1±18.7 | 0.453 | 67.5±19.9 | 62.0±18.6 | 0.085 |
| Emotional well-being | 71.7±18.7 | 71.9±18.5 | 0.934 | 72.7±18.2 | 67.9±19.9 | 0.219 | 72.0±18.5 | 69.1±19.7 | 0.582 | 72.8±18.5 | 70.7±18.7 | 0.472 |
| Social functioning | 84.7±20.6 | 82.3±22.0 | 0.493 | 85.2±20.1 | 77.7±24.4 | 0.135 | 83.8±21.4 | 83.9±19.3 | 0.986 | 84.9±21.5 | 82.8±20.8 | 0.545 |
| Bodily pain | 76.3±23.1 | 73.6±23.5 | 0.493 | **77.5±21.7** | **65.8±27.3** | **0.041** | 76.2±23.3 | 67.1±20.8 | 0.165 | 78.7±21.6 | 72.0±24.4 | 0.077 |
| General health | 55.5±18.2 | 50.6±18.1 | 0.118 | 54.8±18.2 | 48.9±18.2 | 0.126 | 54.6±18.4 | 45.0±15.2 | 0.061 | 56.4±18.5 | 51.0±17.7 | 0.068 |
| Physical component score | **47.7±8.9** | **40.8±12.5** | **<0.001** | **47.3±8.4** | **36.0±15.1** | **0.001** | **46.3±9.6** | **34.3±16.4** | **0.018** | **48.9±7.8** | **41.3±12.2** | **<0.001** |
| Mental component score | 50.5±12.2 | 51.9±11.9 | 0.479 | 51.5±12.1 | 50.6±12.3 | 0.832 | 50.8±12.2 | 53.1±10.5 | 0.501 | 51.1±12.3 | 50.9±11.9 | 0.892 |
| **Disability** | | | | | | | | | | | | |
| Impaired ADL | 1 (1.1%) | 2 (3.6%) | 0.555 | 1 (0.8%) | 2 (7.1%) | 0.090 | 2 (1.5%) | 1 (7.1%) | 0.256 | 1 (1.3%) | 2 (2.7%) | 0.617 |
| Impaired IADL | **4 (4.2%)** | **12 (21.8%)** | **0.001** | **7 (5.7%)** | **9 (32.1%)** | **<0.001** | **10 (7.4%)** | **6 (42.9%)** | **0.001** | **4 (5.3%)** | **12 (16.2%)** | **0.030** |
